# Long-term maintenance of increased exercise involvement following a self-management intervention for housebound older adults with arthritis

**DOI:** 10.1186/1479-5868-4-22

**Published:** 2007-06-04

**Authors:** Kareen Nour, Sophie Laforest, Lise Gauvin, Monique Gignac

**Affiliations:** 1 Centre de recherche et d'expertise en gérontologie sociale (CREGES), CSSS Cavendish-Centre Affilié Universitaire, Montreal, Quebec, Canada; 2 Department of Kinesiology, University of Montreal; Associate Researcher, Groupe de recherche interdisciplinaire en santé (GRIS), University of Montreal, and Centre de recherche et d'expertise en gérontologie sociale(CREGES), CSSS Cavendish-Centre Affilié Universitaire, Montreal, Quebec, Canada; 3 Department of Social and Preventive Medicine, University of Montreal; Associate Researcher, Groupe de recherche interdisciplinaire en santé (GRIS), University of Montreal; Researcher, The Léa-Roback Centre on Social Inequalities of Health in Montreal, Montreal, Quebec, Canada; 4 Health Care & Outcomes Research, University Health Network & Associate Professor, Department of Public Health Sciences, University of Toronto, Toronto, Ontario, Canada

## Abstract

**Background:**

Sustained maintenance of health behaviors is a determinant of successful symptom reduction strategies for older adults with arthritis. This study examined whether or not short-term improvements in exercise involvement were maintained 8 months following a home-based arthritis self-management intervention as well as the moderating role of individual characteristics in the maintenance of behavior change.

**Methods:**

Of the 113 housebound older adult participants at pre-intervention, 97 completed the post-intervention interview, and 80 completed the 8-month post-intervention interview.

**Results:**

Some post-intervention improvements in exercise involvement were maintained 8 months later. More specifically, weekly exercise frequency, particularly regarding walking frequency, and variety of exercise activities were still significantly greater in the experimental group than in the control group 8 months following the completion of the intervention. No moderating influences were observed for any of the individual characteristics.

**Conclusion:**

We conclude that gains in exercise involvement achieved through a self-management intervention can be maintained 8 months following the intervention.

## Background

Community-based arthritis self-management interventions have been shown to have significant short-term benefits on health status [1,2] as well as on adoption and maintenance of health behaviors among older populations with arthritis [1,3-8]. Although regular involvement in health behaviors is recognized as a crucial element in successful symptom-reduction strategies [9,10], very little is known about the extent to which self-management interventions result in sustained behavior change [11-16]. The present follow-up study examined whether or not short-term gains after a home-based self-management arthritis intervention called *I'm Taking Charge of My Arthritis! *[17] were maintained 8 months later. A previous study examining short-term behavior changes following the intervention through a randomized controlled trial (RCT) showed that homebound older adults participants increased frequency of exercise involvement (walking and stretching exercises) as well as variety of exercises [18]. The current follow up study also explored the potential moderating role of participants' socio-demographic [19-21],. psychological [22-25]., and physical characteristics [26] on maintenance of behavior change [27]. This second aim is of particular relevance because examination of the short-term impact of the intervention showed that participants with lower income had smaller intervention gains than more affluent individuals, and those with depression experienced no gains, in contrast to non-depressed participants who showed improvements [18].

The few studies having examined long-term maintenance of intervention effects show mixed results [11-15]. For example, Fries *et al*. [15] showed limited or no long-term maintenance of exercise frequency. Barlow *et al *[14] observed that individuals with arthritis who participated in a community-based self-management intervention successfully maintained changes in communication with physician, use of strategies of distraction and visualization, performance of relaxation and flexibility exercises at 4 and 12 months post-intervention. Conversely, Lindroth *et al*[12] found that among rheumatoid arthritis (RA) participants, frequency of practice was not maintained either at 12 months post-intervention or 5 years later [13]. Finally, the only study evaluating maintenance of changes from a home-based arthritis intervention showed that improvements in frequency of walking and level of household and self-care activities were maintained [16] for RA participants at 12 and 52 weeks post-intervention.

## Methods

Details of the methods and selection of participants have been reported elsewhere [18] but are summarized briefly below.

### Procedures

Eight trained interviewers administered questionnaires to participants in their homes. They were blind to group allocation and to the intervention's specific objectives. Interviews were used to measure all variables and lasted about two hours. Interviews were conducted upon recruitment (baseline), 8 weeks later prior to randomization (pre-intervention), upon completion of the intervention (post-intervention-1) and 8 month post-intervention (post-intervention-2). In most cases (75%), the same interviewer performed all four interviews with participants. The ethics committee of the CSSS Cavendish approved the study and participants signed an informed consent form prior to involvement.

### Participants and design

One hundred and twenty five older housebound adults living with osteoarthritis or rheumatoid arthritis composed the initial sample. Between baseline and pre-intervention measurements, 12 participants dropped out. From the 113 remaining participants, 65 were randomly assigned to an experimental and 48 to a one-year wait list control group. Due to dropout, 97 completed the post-intervention-1 measures (experimental, n = 58; wait list control, n = 39) and 80 completed the post-intervention-2 measures (experimental, n = 50; wait list control, n = 30). Persons dropping-out between the pre-intervention and post-intervention-1 measure (n = 16) reported fewer everyday coping behaviors and higher depression levels than those who persisted. The 17 participants who dropped out between the two post-intervention measures reported less variety in their exercises in comparison to those who persisted in the study. Reasons for dropping out from post-intervention-1 to post-intervention-2 were related to health related problems (e. g., needed hospitalization, deterioration of health status) or to housing (e.g., moving to a seniors residence). To our knowledge, only one participant dropped out due to study burden. Finally, no statistically significant differences in sociodemographic variables were observed between the initial sample of 113 participants and the 80 participants persisting in the study (results not shown).

### Intervention

Participants in the experimental group participated in an intervention called *I'm Taking Charge of my Arthritis ! *[17,28]. This intervention consisted of six sessions of one-hour done in the participant's home by a trained health care practitioner. Each visit included a review of the previous visit, an exploration of a new topic, and the formulation of a new personal contract. A variety of procedures was used to ensure practitioner reliability in intervention implementation [see [18]].

### Post-intervention follow-up

Following the intervention, a subset of the experimental group (n = 29) received social reinforcement through bi-monthly support telephone calls for a period of 6 months whereas the remaining experimental participants (n = 39) did not receive this support. Preliminary analyses indicated that this reinforcement did not have a significant impact on the health and the health behaviors of participants. Therefore, both groups were merged into one experimental group for examination of the effects of the intervention on maintenance. No visits from the interviewer or the interventionists were conducted either in the experimental or control groups. Moreover, no phone calls from the research team to participants were made during this period. Therefore, no actual observations were made and no data were collected in regard to the health or the health behaviors of participants.

### Variables and measures

For the current study, only those outcome variables that showed significant changes immediately following the intervention [18] were examined, namely frequency and variety of exercise involvement. Conceptually this choice is appropriate because the focus of the current study is on maintenance of change rendering examination of variables not showing change incongruous. Empirically, preliminary analyses showed that those variables not influenced by the intervention between baseline and post-intervention-1 remained unchanged 8 months later (at post-intervention-2) (results not shown). Data on socio-demographic characteristics were collected at baseline and information on physical and psychological characteristics was collected at pre-intervention. Data on exercise involvement were recorded at all four measurement times.

#### Outcomes variables

Exercise was categorized into three broad types of activity: walking, stretching, and strengthening. Participants were asked to estimate the number of times per week they performed each activity (from 0 to 7 times/week). A composite of the total weekly occurrence of exercise was calculated by adding the weekly frequencies of the 3 types of activities. Scores could range from 0 to 21 (sum of the 3 activities for 7 days). An index of the variety of activities adopted was computed by dividing the number of exercise activities reported for each type of exercise by the total number of exercises possible and then multiplied by 100 (score varying from 0% to 100%).

#### Confounding variables

Perceived socioeconomic status [29,30], level of education, age, living situation, and gender were self-reported. Type of arthritis was recorded by the case manager. A composite physical factor (from -1 to +1) was created through a factor score resulting from factor analysis of various scales that evaluated pain intensity, fatigue,(0–100 VAS)[13] limitations and stiffness (0–5 WOMAC)[31]. Those scales were used in various study on with arthritis individuals [6,32-34]. Higher scores on the factor reflected greater disability. Finally, a composite psychological factor was created through a factor score resulting from another factor analysis of measures of optimism (1–5 scale) [35], mastery (1–5 scale)[36], and self-efficacy (0–100 SES)[37]. Higher scores on the psychological factor reflected greater psychological health. Depression was evaluated by the CES-D [38]. Participants were dichotomized into depressed (mean scores of 16 or above out of 60) and not depressed.

### Statistical analyses

We applied multilevel modeling techniques to overcome the challenges presented by the data set (e.g., different initial and final sample sizes) and the advantages related to theses techniques (e.g., inclusion of data from all participants collected at any time). The goals of the data analysis were to determine if the intervention resulted in a long-term maintenance of (a) the weekly frequency of exercise (strengthening, stretching, and walking) activities and (b) the variety of exercises, despite controlling for potential confounding effects. A secondary aim consisted of exploring whether or not there were any moderating influences of individual characteristics.

Analyses were performed with SPSS (version 10) and HLM 5.04 (Hierarchical Linear Modeling, Scientific Software International, Chicago, IL). A first set of analyses included parameters operationalizing time and group membership in order to explore whether or not there were changes across time and as a function of group membership. Second and third sets of analyses were performed by adding possible confounding variables to the models in order to examine whether or not intervention effects were maintained despite controlling for possible confounders. For moderating effects, interaction terms between group membership and depression or socioeconomic status were created and entered into the model both with and without control for confounding variables. Significant interactions were plotted to allow for interpretation.

## Results

Of the 113 participants (M = 77.7 years, SD = 10.3) at pre-intervention, 90% were women, with less than 9 years of education (47%). About 71% lived alone. Sixty-three percent of participants had been diagnosed with OA and 83% reported that they were financially comfortable. Participants who completed the post-intervention-2 measurement (n = 80) were, on the whole, similar to participants who had been randomized at pre-intervention (n = 113). They were aged 76.6 years olds (SD = 11.0), 89% were women and 74% lived alone. All participants' characteristics at pre-intervention are presented in Table 1. A more exhaustive descriptive profile of the sample is available elsewhere [8,39].

**Table 1:** Demographic characteristics of participants.

	*Pre-intervention participants*	*Post-intervention participants*	*Post-intervention2 participants*
	
	*N = 113 M(SD) or %*	*N = 97 M(SD) or %*	*N = 80 M(SD) or %*
Age (years)	77.70 (10.31)	77.27 (10.67)	76.61 (10.99)
Women (%)	90.3	90.7	88.8
Poor/very poor self-reported economic statues	18.6	17.5	17.5
Education (years)	9.33 (4.11)	9.21 (4.04)	9.32 (3.81)
Living alone (%)	70.8	71.1	73.8
Osteoarthritis	62.8	62.9	63.8
No depression (> 16 on 60)	41.4	42.7	42.3

Table 2 shows the observed values and relative increases in outcome measures for experimental and control groups at different times. Results of the multilevel modeling analyses are described below and appear in Table 3.

**Table 2:** Observed Means and Relative Increases in Outcome Variables at Different Times for each Group

Measurement time	Baseline			Pre-intervention		Post-intervention-1		Post-intervention-2		*% increase (post2-post1/post1)*	
					
Outcome variables	Max	*Exp.*	*Control*	*Exp.*	*Control*	*Exp.*	*Control*	*Exp.*	*Control*	*Exp.*	*Control*
Group											
						
Variety of exercises	100	47	49	47	38	63	41	60	43	-4.76	4.87
Occurrence of exercises (times/week)	21	7.35	7.12	6.48	4.79	10.02	5.64	7.04	6.17	-29.74	9.40
- stretching (times/week)	7	2.85	2.73	2.56	2.06	4.57	2.30	2.80	3.03	-38.73	31.74
- walking (times/week)	7	2.26	2.83	2.04	1.77	2.57	1.72	2.76	1.93	7.40	12.21
- strengthening (time/week)	7	2.25	1.56	1.87	.96	2.88	1.61	1.48	1.90	-48.61	18.01

**Table 3:** Hierarchical Linear Models Analyses Predicting Exercise Change and Maintenance following the Intervention

		Baseline			Pre-intervention			Post-intervention 1				Post-intervention 2			
					
Within-Subject Fixed Effects		γ_*ij*_	*Coeff.*	*SE*	γ_*ij*_	*Coeff.*	*SE*	γ_*ij*_	*Coeff.*	*SE*	*p*	γ_*ij*_	*Coeff.*	*SE*	*p*^*a*^
				
Variety of exercises	Intercept	γ_00_	.51	.04	γ_10_	-.14	.05	γ_20_	-.10	.05	.08	γ_30_	-.10	.05	.12
	Group	γ_01_	-.03	.06	γ_11_	.13	.06	γ_21_	.24	.06	.**00**	γ_31_	.22	.07	**.00**
Occurrence of exercise	Intercept	γ_00_	7.78	.83	γ_10_	-3.00	.86	γ_20_	-2.33	0.92	.01	γ_30_	-2.31	1.02	.02
	Group	γ_01_	-.97	1.10	γ_11_	2.40	1.13	γ_21_	5.23	1.19	**.00**	γ_31_	2.54	1.30	**.05**
- stretching	Intercept	γ_00_	3.12	.38	γ_10_	-1.06	.42	γ_20_	-.87	.45	.06	γ_30_	-.45	.50	.37
	Group	γ_01_	-.40	.50	γ_11_	.76	.56	γ_21_	2.63	.59	**.00**	γ_31_	.44	.64	**.50**
- walking	Intercept	γ_00_	2.67	.36	γ_10_	-.90	.42	γ_20_	-.99	.45	.03	γ_30_	-1.12	.50	.03
	Group	γ_01_	-.56	.48	γ_11_	.79	.56	γ_21_	1.35	.59	.**02**	γ_31_	-1.59	.64	**.01**
- strengthening	Intercept	γ_00_	2.00	.36	γ_10_	-1.00	.39	γ_20_	-.39	.41	.30	γ_30_	-.69	.46	.14
	Group	γ_01_	-0.01	.47	γ_11_	1.09	.51	γ_21_	.91	.53	**.10**	γ_31_	.39	.59	**.51**

Statistical significance of coefficient γ_31 _is indicative of maintenance effects using the following model:

Level 1 model: Outcome = β_0j _+ β_1j_X_1 _+ β_2j_X_2 _+ β_2j_X_2_+ r_ij_

Level 2 model: T_1 _β_0j_= γ_00 _+ γ_01 _Gr_j _+ u_oj_

T_2 _β_1j_= γ_10 _+ γ_11 _Gr_j_

T_3 _β_2j_= γ_20 _+ γ_21 _Gr_j_

T_4 _β_2j_= γ_30 _+ γ_31 _Gr_j_

where i: 1 ....N individuals ; r_ij: _level 1 error term ; γ_ij _: coefficients for parameters ; u_oj: _level-2 random effect ; β: coefficient ; Gr: group membership (1 = experimental, 0 = control) ; X_1_: dummy variable (1 = pre-intervention, 0 = otherwise) ; X_2_: dummy variable(1 = post-intervention-1, 0 = otherwise); X_3_: dummy variable(1 = post-intervention-2, 0 = otherwise)

Meaning of Coefficients:

γ_00 _= Predicted value in outcome variable at baseline for participants in control group

γ_01 _Gr_j _= Predicted difference in outcome variable at baseline for participants in experimental group

γ_10 _= Predicted change in outcome variable from baseline to pre-intervention for participants in control group

γ_11 _Gr_j _= Predicted difference in change in outcome variable from baseline to pre-intervention for participants in experimental group

γ_20 _= Predicted change in outcome variable from baseline to post-intervention1 for participants in control group

γ_21 _Gr_j _= Predicted difference in change in outcome variable from baseline to post-intervention-1 for participants in experimental group

γ_30 _= Predicted change in outcome variable from baseline to post-intervention-2 for participants in control

γ_31 _Gr_j _= Predicted difference in change in outcome variable from baseline to post-intervention-2 for participants in experimental group.

^*a *^significance of the changes that had occurred between post-intervention-1 and post-intervention-2

In terms of the first objective, namely to examine whether or not short-term gains after a self management arthritis intervention were maintained at 8 months, results showed that the weekly occurrence of exercise was maintained 8 months after completion of the intervention (p = .05) even though data show a decrease in exercise involvement across time for experimental group participants. That is, experimental participants decreased their mean weekly occurrence of exercise from 10.02 times per week immediately following the intervention to 7.04 times per week at 8-months after the intervention while control participants' frequency of involvement in exercise changed from 5.64 times per week to 6.17 times per week. Nevertheless, multilevel modeling analyses showed that the decrease in the experimental group was not large enough to obviate between-group differences, suggesting the maintenance of effects (p = .05) after the end of the program.

The variety of exercises were maintained 8 months after completion of the intervention (p = .05). Although experimental participants decreased slightly their mean variety of exercises from 63% immediately following the intervention to 60% at 8-months post-intervention, control participants' increased their variety of exercises from 41% to 43%. Multilevel modeling analyses showed that the variety of exercises was maintained across time (p = .01).

Analyses of the three types of exercises showed that the weekly frequency of walking activity was maintained (p = .05), but that the frequency of stretching exercises was not (p = .50). In fact, the mean walking frequency of experimental participants increased in the 8 month period from 2.57 times per week to 2.76 times per week while the frequency of walking of control group participants increased from 1.72 times per week to 1.93 times per week. Multilevel modeling analyses indicated that walking frequency was maintained across time and that differences between the groups were still statistically significant (p =.01). However, during the same period, there was a significant decrease in experimental participants' frequency of stretching exercises from 4.57 times per week to 2.80 times per week. As a result, frequency of stretching exercises was not maintained (p = .50). The weekly frequency of strengthening exercises, which did not change immediately following the intervention, remained unchanged in long term (p = .51). These findings were not altered after controlling for confounding variables.

In terms of the second goal, namely to explore the potential moderating role of participants' sociodemographic, psychological., and physical characteristics on maintenance of behaviour change, no significant results were obtained as none of the interaction terms achieved statistical significance. The findings suggest the absence of any moderating influences of age, gender, education, living arrangements, physical symptoms and disability, and psychological health. That is, changes were maintained across a diverse range of groups.

## Discussion

This study examined whether or not short-term improvements in exercise involvement were maintained 8 months following a home-based arthritis self-management intervention as well as the moderating role of individual characteristics in the maintenance of behavior change. Results showed that behavior changes observed immediately after the self-management intervention for variety and frequency of exercise were maintained 8 months following completion of the intervention despite apparent decreases in exercise involvement. Furthermore, long-term maintenance was not moderated by individual characteristics. Among the three subtypes of exercises assessed, only changes in walking remained statistically significant with participants reporting walking even more frequently 8-months after the program.

These results are congruent with some studies on maintenance of involvement in exercise after self-management interventions [4,16]. Given the rigorous study design, we propose that maintenance is related directly to the "quality of the content" of the intervention [40-42]. First, participants were strongly encouraged to take daily walks in their homes and immediate surroundings. They were shown, during the intervention, that walking is an "easy-to-do" exercise that can be performed anywhere, at any time. This demonstration and the verbal persuasion may have supported participants in their maintenance of exercise. Secondly, participants highly valued "increasing walking frequency or distance" in the personal contracts implemented during the intervention. Given that these contracts stay with participants after the intervention as "reminder" of the importance of staying active, they might have supported their exercise maintenance. Finally, during the intervention, participants received an exercise hand-out with examples of stretching and strengthening exercises. Even though these types of exercises were not maintained across time, this sheet might also been an exercise "reminder" which might have influence exercise maintenance. Together, the hand-out, the encouragement received, and the personal contract may have had a long-term impact on motivating people to stay active, to get out of the home, and even to use resources to exercise.

However, given that maintenance of intervention changes are usually precarious, other strategies could be implemented. First, even if social reinforcement by interventionists did not have significant impact on exercise maintenance, we propose that such reinforcement should be delivered by the practitioner intervening with participants because interventionists are the ones who know and understand participants and with whom the participant shares a bond of trust. Secondly, new exercise information could be sent periodically to participants in order to strengthen the value of staying physically active as well as to provide a new series of home exercises. Finally, considering the absence of maintenance of stretching and strengthening activities, the new version of the *I'm Taking Charge of My Arthritis! *intervention, now includes additional hand-out exercise sheets. In fact, it is possible that participants became bored with repeating the same six stretching and strengthening activities provided on the exercise hand-out and decided to walk instead because it is easier and more pleasant. These changes may help future participants maintain intervention benefits.

In addition to the previous results, it also interesting to note that even though short-term changes in weekly exercise frequency were moderated by economic status and depression, no individual characteristics played a moderating role in the maintenance process. This finding is in contrast to others studies [41] who highlight functional limitations, comorbid situation, depressives symptoms, gender [43,44], self-efficacy, and perceived behavioral control [45-47] as predictors of maintenance of health behavior. Such results have important implications for setting and timing of intervention. In fact, our long-term results imply that when interventions result in changes, everyone, regardless of their individual characteristics can maintain them.

Despite these findings and interpretations, several limitations of the study should be mentioned. Participants may have demonstrated social desirability bias in responding to interviews and thus reported more exercise than they actually performed. Similarly, other variables not measured in our study (i.e., social support by the family or involvement in other activities) might explain greater maintenance of exercise involvement. Furthermore, limitations to the RCT were discussed elsewhere [18] and included the self-reported measures and the relatively small number of participants. Additional limitations are the long-term follow-up wherein no experimental manipulation was done between post-intervention-1 and post-intervention-2, which limits interpretation of the findings. Furthermore, the sample of the current study was composed predominantly of females and thus calls into question its generalizability. However, as other studies have shown [12-15], females are more likely to suffer from arthritis than males (54% versus 37% in Canada for older adults) thus likening the study context to a real-world situation.

Nevertheless, considering the frail and housebound nature of the target population and the large variety of positive impacts exercise can have on their health, the current findings remain encouraging. Moreover, the low to moderate levels of pre-intervention exercise of this population and the significant improvements and some long-term maintenance of exercise behaviors support the notion that providing such interventions on a wider scale could have significant benefits in the management of disability. Interventions at home may facilitate learning and adoption of health behaviors.

To our knowledge, this is the only study conducted entirely on housebound older adults with arthritis for the purpose of evaluating a home-based self-management intervention. Health promotion interventions such as *I'm Taking Charge of My Arthritis! *can support the plight of homebound older adults with arthritis who must deal with arthritis symptoms on a daily basis.

## Competing interests

The author(s) declare that they have no competing interests.

## Authors' contributions

Kareen Nour and Sophie Laforest conceptualized the research project, participated in the design of the study and wrote the article. Kareen Nour further developed the ideas and the statistical analysis and coordinated the gathering of data. Sophie Laforest, as principal researcher of the project *I'm Taking Charge of My Arthritis!*, undertook the general supervision of the project and actively participated in each step. Lise Gauvin guided the statistical analysis and contributed to the conceptualization and write-up of the manuscript. Monique Gignac made a significant contribution to writing of the article, through a critical review of the intellectual content. All authors read and approved the final manuscript.
